# Refractory IgG4−related skin disease successfully treated with intravenous methylprednisolone and baricitinib

**DOI:** 10.1016/j.jdcr.2026.07.049

**Published:** 2026-08-01

**Authors:** Jacqueline Jiang, Kate Simpson, Christopher McCormack, Emma Veysey, Amanda M. Saracino

**Affiliations:** a Department of Dermatology, St Vincent’s Hospital, Melbourne, Australia; b Skin Health Institute, Melbourne, Australia; c Department of Dermatology, Western Health, Melbourne, Australia; d Department of Dermatology, Peter MacCallum Cancer Centre, Melbourne, Australia; e Department of Dermatology, The Royal Women’s Hospital, Melbourne, Australia; f Department of Medicine, University of Melbourne, Australia; g Division of Rheumatology and Connective Tissue Diseases, University College London, London, United Kingdom

**Keywords:** baricitinib, fibrosis, IgG4–related disease, Janus kinase inhibitors, skin diseases

IgG4**−**related disease (IgG4-RD) is a systemic condition characterized by chronic, relapsing fibroinflammatory lesions that can occur in multiple organs and cause progressive organ damage.^1^ Skin manifestations of IgG4-RD are uncommon, but they can present as erythematous pruritic plaques, indurated subcutaneous nodules or papules, mimicking other inflammatory or infiltrative diseases, or malignancies.^2^, ^3^, ^4^ Histopathological findings in cutaneous IgG4-RD include dermal or subcutaneous lymphoplasmacytic infiltration, increased infiltrating IgG4**−**positive plasma cells, obliterative phlebitis, and storiform fibrosis.^2^, ^3^, ^4^ Elevation of the serum IgG4, which ordinarily constitutes about 5% of the total IgG, is a useful marker for diagnosis (sensitivity 86% and specificity 90%).^5^ However, the rarity of cutaneous manifestations of IgG4-RD and nonspecific clinical presentation poses a diagnostic challenge.^2^^,^^3^ Although there are validated classification criteria for IgG4-RD, these criteria exclude patients with IgG4**−**related skin-only disease.^1^

The pathogenesis of IgG4-RD is not fully understood, but current treatments broadly attempt to reduce inflammation and secondary tissue fibrosis. Management guidelines are lacking and therapeutic efficacy data are limited. Glucocorticoids are typically first line.^2^^,^^4^ However, there is a high risk of disease relapse with steroid tapering and treatment morbidity with longer-term maintenance therapy.^2^^,^^4^ Steroid-sparing agents such as azathioprine, mycophenolate mofetil, methotrexate, cyclophosphamide, and rituximab have been reported with some success in patients with inadequate response, relapse, or intolerance to medium-to-high doses of glucocorticoids.^2^^,^^4^

We report a case of cutaneous IgG4-RD refractory to multiple conventional therapies and rituximab, successfully treated with intravenous methylprednisolone followed by baricitinib.

## Case presentation

A 58-year-old woman presented with recurrent painful, firm, and indurated plaques on the right breast (Fig 1) and subcutaneous nodules on the shoulder and buttocks. There were no associated respiratory, gastrointestinal, or constitutional symptoms.

**Fig 1:**
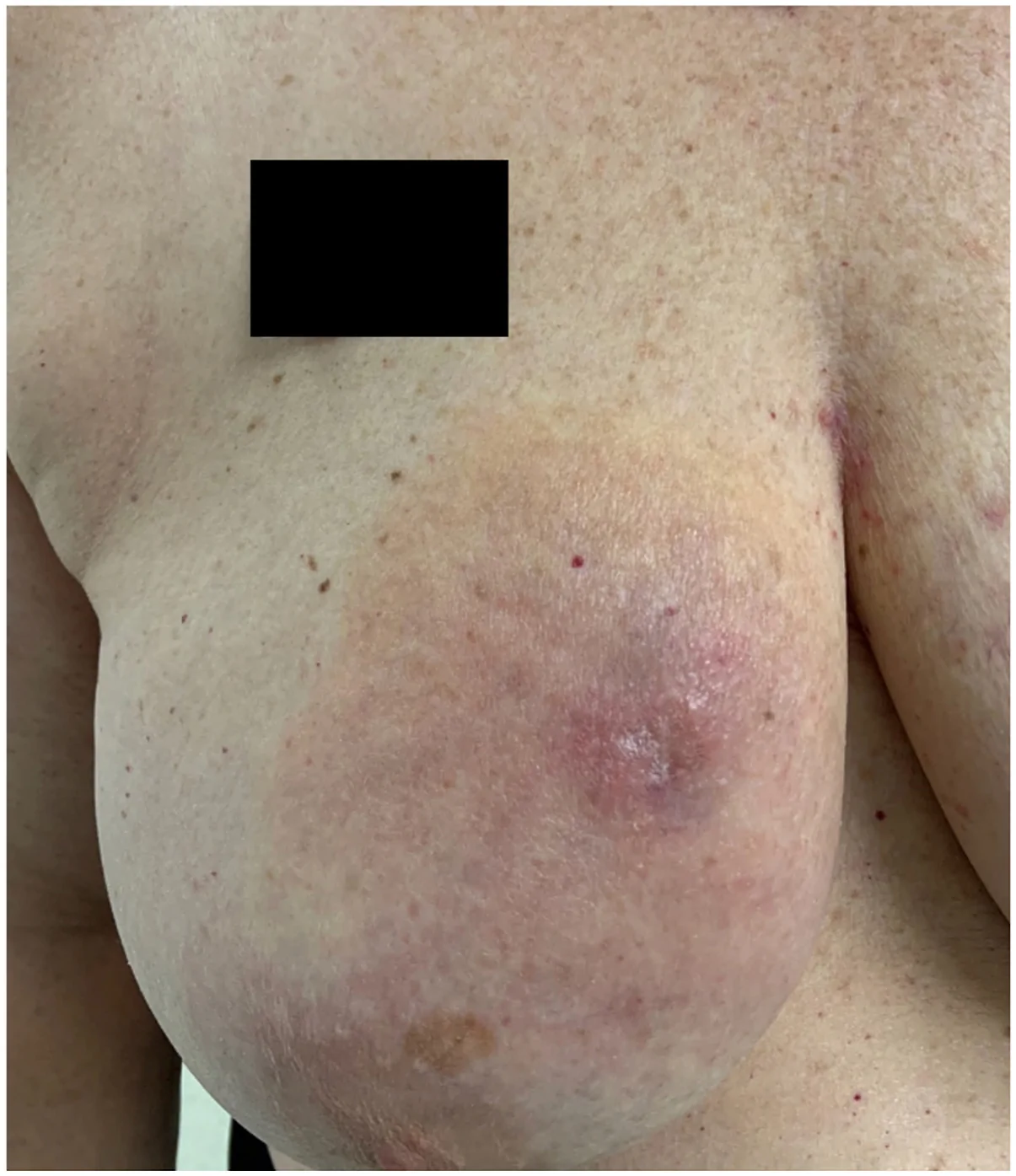
Painful, firm, indurated plaque on the right breast (July 2024).

Excisional biopsies of the plaque and subsequent recurrent lesions demonstrated lymphocytic lobular panniculitis, dense plasma cell−rich infiltrates, increased IgG4 plasma cells (20-40 per high-power field), and IgG4:IgG ratio >40%, and increased collagen bundles in the upper- to mid-dermis.

Serologic testing showed IgG4 elevated to 2.54 g/L (reference range, 0.03-2.01 g/L). Other tests, including full blood count, antinuclear antibody, extractable nuclear antigens, double-stranded DNA, rheumatoid factor, anticyclic citrullinated peptide, antineutrophil cytoplasmic antibodies, erythrocyte sedimentation rate, human leukocyte antigen-B27, complement proteins, angiotensin-converting enzyme, α-1-antitrypsin, κ–free light chain, protein electrophoresis, lactate dehydrogenase, β-2 microglobulin, peripheral blood lymphocyte markers, immunoglobulins, mast cell tryptase, and preimmunosuppressive screens, were unremarkable. Whole-body positron emission tomography, breast magnetic resonance imaging, and thoracoabdominopelvic computed tomography scans were negative for mass or diffuse organ involvement, malignancy, lymphadenopathy, and lymphoproliferative disorder.

The patient was diagnosed with IgG4**−**related sclerodermoid plaques without other organ involvement. The plaques and subcutaneous nodules recurred after excisional biopsies and surgical excision, and they continued to enlarge (Fig 2, *A*) despite multiple systemic therapies, including hydroxychloroquine (200 mg twice a day for 6 months), methotrexate (20 mg weekly for 10 weeks), dapsone (50 mg daily for 11 months), rituximab (2 cycles of 1 g at 6-month intervals), and intralesional triamcinolone (10 mg/mL). A course of oral prednisolone 37.5 mg daily provided partial response but was poorly tolerated due to insomnia.

**Fig 2:**
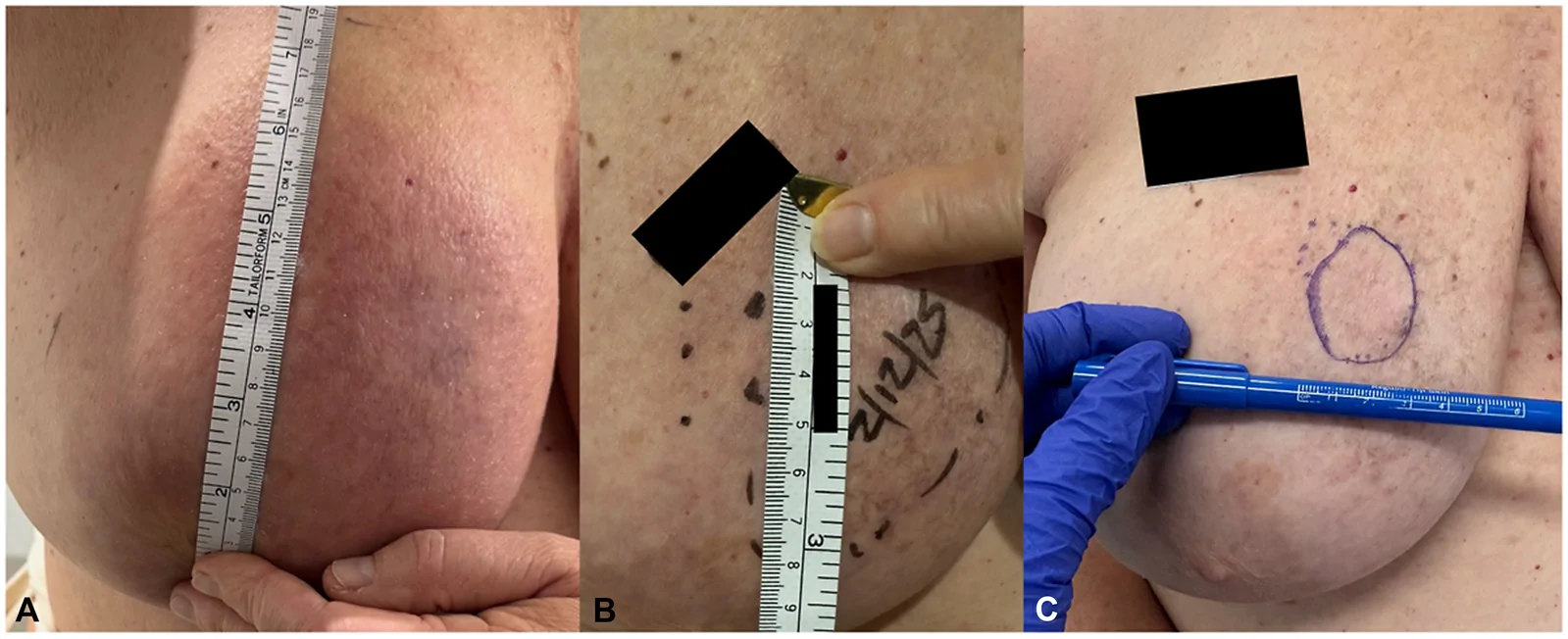
A, Enlargement of plaque on the right breast to 18.5 cm × 19 cm despite multiple conventional systemic therapies (hydroxychloroquine, methotrexate, dapsone, and rituximab) (April 2025). **B,** 7 cm × 8 cm plaque on the breast after 6 months of monthly methylprednisolone and baricitinib 4 mg daily combination therapy. **C,** 3 cm × 4 cm plaque on the breast after a further 7 months of baricitinib and slow prednisolone wean (from 5 mg to cessation).

Five years after symptom onset and following multiple failed therapies, the patient was commenced on monthly intravenous methylprednisolone (500 mg daily for 3 consecutive days, then 1 g monthly) and baricitinib 4 mg daily. After 6 months of methylprednisolone and baricitinib, there was a reduction in erythema, firmness, and size of the breast plaque from 18.5 cm × 19 cm to 7 cm × 8 cm (Fig 2 *A, B*), and reduction in size and firmness of the subcutaneous nodules. Methylprednisolone was stopped after 6 months, and baricitinib was continued as maintenance therapy at 3.4 mg daily, with prednisolone 5 mg daily (weaned by 1 mg/month). Seven months later, the serum IgG4 level normalized to 0.89 g/L; there was ongoing improvement in the firmness of the breast plaque, which had reduced to 3 cm × 4 cm (Fig 2, *C*), and complete resolution of the subcutaneous nodules.

## Discussion

Treatment of IgG4-RD is challenging. There is no consensus for managing cutaneous manifestations of IgG4-RD, and current evidence for treatment is limited to case reports and small case series. Glucocorticoid−based first-line regimens are typically used for cutaneous and multiorgan IgG4-RD manifestations with good initial response rates, but relapse rates are high, especially with tapering.^2^^,^^4^^,^^6^ In a systematic review of 36 cases of cutaneous IgG4-RD with treatment data, complete resolution without relapse was uncommon (11.1%).^4^ One-third of patients (33.3%) demonstrated no response or relapsed with glucocorticoid tapering, and less than half of cases (47.2%) demonstrated complete response after relapse despite slow tapering of systemic steroids or switching to alternative agents.^4^ Managing IgG4-RD can also be complicated by steroid-related adverse events and comorbidities limiting treatment tolerability.^2^^,^^6^ Our patient did not tolerate oral glucocorticoids and failed to respond to several conventional and targeted steroid-sparing agents. Thus, the lack of established effective therapies for cutaneous IgG4-RD called for investigation into other potential alternative agents.

Janus kinases (JAKs) are a family of nonreceptor cytoplasmic tyrosine kinases that mediate the intracellular signaling of downstream proinflammatory and profibrotic cytokine receptors. JAK inhibitors have been proposed as a potential treatment target for IgG4-RD.^6^ The expression of IgG4 is upregulated by T helper 2 cytokines, interleukin 4 and interleukin 13; and regulatory T-cell cytokines, interleukin 10, and transforming growth factor β.^7^^,^^8^ Transforming growth factor β is also known to induce fibrosis and may contribute to fibrotic activity in IgG4-RD.^7^ Tofacitinib, a selective JAK1 and JAK3 inhibitor, has been used to wean systemic glucocorticoids and maintain disease remission in systemic IgG4-RD.^9^ Baricitinib, a selective JAK1 and JAK2 inhibitor, has been shown to inhibit pro-inflammatory fibroblast activation^10^ and alleviate skin fibrosis in cutaneous fibrotic disorders such as generalized morphea/eosinophilic fasciitis, and sclerodermoid graft versus host disease.^8^ Hence, in the absence of contraindications, baricitinib was a suitable next-line treatment option for our patient. Our case demonstrates cutaneous IgG4-RD successfully treated with a JAK inhibitor and systemic corticosteroids.

In conclusion, treatment of cutaneous IgG4-RD is challenging and poorly evidenced. Chronic fibroinflammatory lesions can lead to significant disfigurement and pain, as seen in our patient. In this case, combination intravenous pulsed methylprednisolone and baricitinib led to significant improvement, and disease remission was maintained with baricitinib. This case highlights the antifibroinflammatory potential and promise of JAK inhibition as a therapeutic agent for patients with cutaneous IgG4-RD.

## Conflicts of interest

None disclosed.
